# COVID-19 Diagnosis Using Capsule Network and Fuzzy *C*-Means and Mayfly Optimization Algorithm

**DOI:** 10.1155/2021/2295920

**Published:** 2021-10-19

**Authors:** Ali Farki, Zahra Salekshahrezaee, Arash Mohammadi Tofigh, Reza Ghanavati, Behdad Arandian, Amirahmad Chapnevis

**Affiliations:** ^1^Department of Information Technology Engineering, Industrial and Systems Engineering Faculty, Tarbiat Modares University, Tehran, Iran; ^2^Florida Atlantic University, College of Engineering and Computer Science, Boca Raton, Florida 33431, USA; ^3^Department of General Surgery, School of Medicine, Shahid Beheshti University of Medical Sciences, Tehran, Iran; ^4^Department of Chemical and Petroleum Engineering, Sharif University of Technology, Tehran, Iran; ^5^Department of Electrical Engineering, Dolatabad Branch, Islamic Azad University, Isfahan, Iran; ^6^Department of Computer Engineering and Information Technology, Amirkabir University of Technology, Tehran, Iran

## Abstract

The COVID-19 epidemic is spreading day by day. Early diagnosis of this disease is essential to provide effective preventive and therapeutic measures. This process can be used by a computer-aided methodology to improve accuracy. In this study, a new and optimal method has been utilized for the diagnosis of COVID-19. Here, a method based on fuzzy *C*-ordered means (FCOM) along with an improved version of the enhanced capsule network (ECN) has been proposed for this purpose. The proposed ECN method is improved based on mayfly optimization (MFO) algorithm. The suggested technique is then implemented on the chest X-ray COVID-19 images from publicly available datasets. Simulation results are assessed by considering a comparison with some state-of-the-art methods, including FOMPA, MID, and 4S-DT. The results show that the proposed method with 97.08% accuracy and 97.29% precision provides the highest accuracy and reliability compared with the other studied methods. Moreover, the results show that the proposed method with a 97.1% sensitivity rate has the highest ratio. And finally, the proposed method with a 97.47% *F*1-score rate gives the uppermost value compared to the others.

## 1. Introduction

In recent decades, several new diseases have emerged in different geographical areas with pathogens including the Ebola virus, Zika virus, NIPA virus, and coronaviruses. Recently, a new type of pathological infection has emerged in Wuhan, China. The new strain is severe acute respiratory syndrome 2 (SARS-CoV-2), which causes Coronavirus Disease 2019 (COVID-19).

Following the increase in the number of patients, the Chinese public clinical and scientific associations reacted quickly to allow the new virus to be identified promptly, and the viral gene sequence to be identified and distributed to other countries around the world. Following extensive research on January 30, 2020, the World Health Organization (WHO) declared the prevalence of public health emergencies to be an international concern [1].

By increasing the extension of this disease, researchers have worked on different methods for early detection of this case at least for minimizing the outbreak. People with suspected COVID-19 should determine as soon as possible if they are infected [2]. Therefore, they should quarantine themselves, receive medical treatment, and inform and warn their relatives. One of the most popular and less harmful imaging methods for diagnosis of this area is chest X-ray imaging. Chest X-ray images are images that use small doses of ionizing radiation to take pictures of the inside of the body called radiographs [3]. The X-rays can help physicians in different cases such as bone fractures, dislocations, or joint inflammation, abdominal pain, and also some cancer cases [4]. Based on this fact, lots of researchers go through work on using chest X-ray imaging for the diagnosis of COVID-19.

Pereira et al. [5] proposed a method for the identification of COVID-19 in chest X-ray images using hierarchical and flat classification scenarios. They used hierarchical and multiclass learners for disease identification. COVID-19 texture was also explored from the chest X-ray images of pneumonia.

Minaee et al. [6] proposed another method for COVID-19 detection from radiology images. The method was performed on chest X-ray images from publicly available datasets. The images were injected to train four general convolutional neural networks, containing ResNet50, ResNet18, SqueezeNet, and DenseNet-121 for COVID-19 diagnosis in chest X-ray images. The method assessed the models on the residual images, and most of the networks presented high sensitivity and specificity ratios. Even though the efficiency was so hopeful, they presented that more examinations were needed to provide a more consistent estimation.

Rasheed et al. [7] proposed a different methodology for the diagnosis of COVID-19 from chest X-ray images. They used two widely used classifiers including logistic regression (LR) and convolutional neural networks (CNN). They also used the principal component analysis (PCA) to decrease the complexity of the system and to increase the speed of the system. They utilized an online available dataset incorporating GAN to have 500 X-ray images. The final results showed high accuracy for the proposed system.

Elaziz et al. [8] proposed another diagnosis system for the detection of COVID-19 from chest X-ray images. They extracted features from the input images based on fractional multichannel exponent moments (FrMEMs). Then, parallel computing was used for speeding up the system. Then, a modified manta-ray foraging optimization algorithm was used to select the main features. The method was assessed based on the COVID-19 X-ray dataset. The proposed method provided high accuracy for the datasets.

Rehman et al. [9] proposed another computer-aided method for fast detection of COVID-19 based on a convolutional neural network (CNN). The method used the residual neural network (ResNet50) for the purpose. For validating the proposed method, it is performed on a dataset containing X-ray images. Simulation results showed about 98% accuracy for the proposed method.

As can be observed from the literature, different techniques based on chest X-ray images have been proposed for the proper diagnosis of COVID-19. The main objective of this paper is to present another optimal methodology to provide a diagnosis system with higher accuracy. The key purpose is to deliver a new precise computer-aided diagnostic system for COVID-19 diagnosis to help physicians for the detection of COVID-19. Here, we utilized a new optimal machine learning-based approach for the computer-aided diagnosis of COVID-19. This study employs an optimal configuration for proper diagnosis of COVID-19 based on an optimized fuzzy *C*-ordered means (FCOM) and an improved version of the enhanced capsule network (ECN). The ECN is modified by using the mayfly optimization (MFO) algorithm. The final results show well results for the proposed method.

## 2. Database Preprocessing

The present study proposed a new method based on a clustering approach to provide a proper automatic segmentation system for COVID-19 diagnosis. More specifically, a new optimized fuzzy *C*-means method (FCM) has been proposed based on a newly developed version of a new metaheuristic algorithm to offer a system with higher efficiency.

However, the traditional clustering methods such as *K*-means clustering make partitions, wherein each cluster includes just one pattern (a set including accurate and crisp values), fuzzy clustering spreads this technique more to give or associate the present patterns in an image with the data clusters of the image based on a membership function. The fuzzy *C*-means contains a “soft clustering” methodology and delivers a precise calculation for the cluster membership and is utilized successfully for image clustering applications, especially in medical imaging.

**Figure 1:**
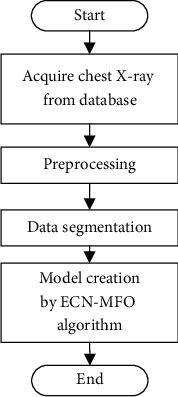
The graphical abstract of the proposed method.

The proposed developed FCM is then used as a new method for segmentation of the chest X-ray of COVID-19. The system classification has been accomplished according to the enhanced capsule network (ECN). The method is based on using deep learning including multiple stages of receipt of raw data supposed as a beginning stage, and the model classification presentation has achieved at the final stage. The graphical abstract of the system is provided in Figure 1.

### 2.1. Dataset Description

**Figure 2:**
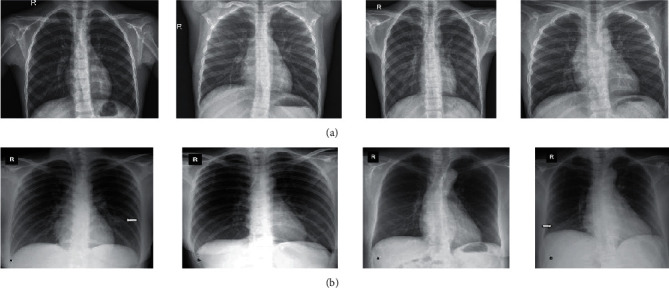
Some examples of the chest X-ray images collected from these datasets: (a) normal case and (b) COVID-19 case.

The method of authentication is proposed based on a standard test case of the COVID-19 dataset. Several datasets have been proposed for the diagnosis of COVID-19. The presented study uses three datasets including a popular resource collected by the Renmin Hospital of Wuhan University and two affiliated hospitals, Sun Yat-sen Memorial Hospital and the Third Affiliated Hospital of the Sun Yat-sen University in Guangzhou with 12 and 76 patients [10].  Figure 2 shows some examples of the chest X-ray images collected from these datasets.

### 2.2. Data Normalization

Sometimes, the raw data we have for analysis is not suitable for a group of statistical tests and to be able to use this category of statistical tests and to increase the accuracy of the analysis, we have to make changes in the raw data. One of these changes is called data conversion. Data conversion is a mathematical method used to modify variables that do not follow the statistical assumptions of normality, linearity, and uniform scattering, or have patterns with unusual outliers.

Among data conversion methods, data normalization has high efficiency. Normalization has different meanings in statistics, the simplest use of which is to normalize data or normalize variables, and is a method that puts data in the same domain when they are not [11]. In other words, a data miner may encounter situations where the properties of the data include values that are in different ranges or domains. These large-value features may have a much greater effect on the cost function than low-value features. This problem will be solved by normalizing the properties so that their values are in the same range [12]. In constructing a metamodel from the data, before model training begins, the data is subdivided into its largest corresponding values to be normalized to values between zero and one scales, to minimize the effect of the absolute scale and have almost all inputs in the same range.

The min-max method is one of the popular and simplest normalization methods in medical imaging [13]. Based on this method, over and above the unifying data scale, the data changing edges will be distributed in the range between 0 and 1. By assuming attribute *X*, such that it has a mapping from the data set between *X*_min_ and *X*_max_, the min-max normalization (*X*_norm_) will be achieved by the following [14]:

(1) $$\begin{array}{c} X_{\mathrm{norm}}=\frac{X-X_{\min}}{X_{\max}-X_{\min}}. \end{array}$$

### 2.3. Contrast Enhancement

**Figure 3:**
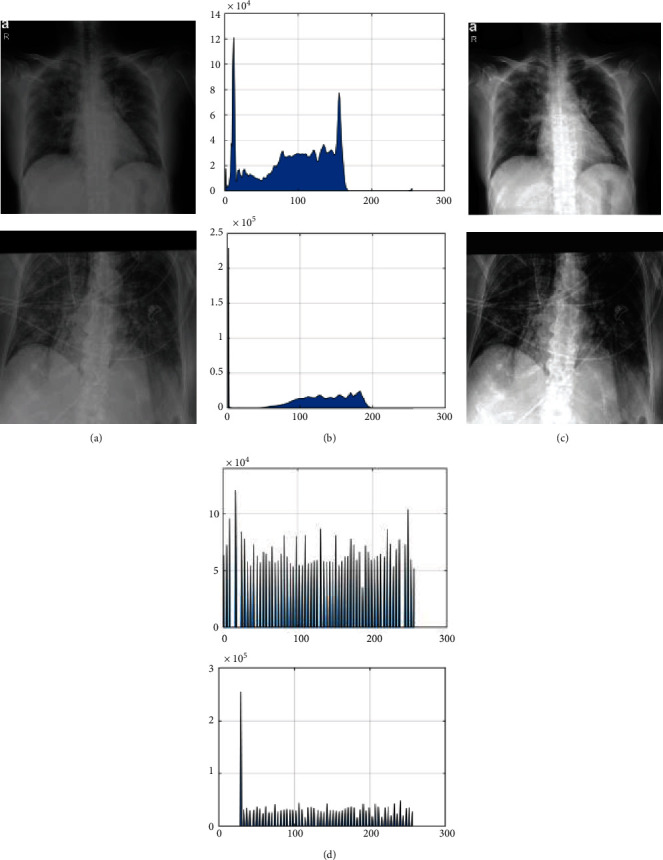
Some examples of the image processing applied to the input images: (a) input image, (b) histogram of (a), (c) image after preprocessing, and (d) histogram of (c).

Another preprocessing step to improve the image quality is contrast enhancement. The image contrast enhancement, especially in medical images, is an important issue that can be improved the accuracy of the system in a sensible term. This process can increase the contrast between different considered objects to simplify the next segmentation steps. In the present study, contrast enhancement has been used on COVID-19 chest X-ray images to highlight the significant areas with keeping the other areas fixed. The study uses a 16-bit lookup table for this purpose that is then stored on a disc. This technique can be mathematically formulated as follows [15]:

(2) $$\begin{array}{c} B_{\text{hist}}=\frac{A_{\text{hist}}-\text{Mi}{\text{n}}_{\text{hist}}}{\text{Ma}{\text{x}}_{\text{hist}}-\text{Mi}{\text{n}}_{\text{hist}}}, \end{array}$$

where  Min_hist_ and Max_hist_ represent the lowest and the highest levels of the gray magnitudes of the main image histogram, respectively, and *A*_hist_ and *B*_hist_ represent the input and the output images before and after contrast enhancement, respectively.  Figure 3 shows some examples of the image processing applied to the input images.

## 3. Mayfly Optimization (MFO) Algorithm

Mayfly is a tiny, fragile, and soft-body insect that has more than 3100 types around the world. However, this insect needs almost one year to birth; it dies after a maximum of 1 day of living. The main target of their birthing is mating. Most of them even do not bother themselves with feeding. The mayfly swarms for mating usually include several males from a few to hundreds of individuals, which are about 1 m to 4 m above the ground for about 1.5 hours to 2 hours in the early morning. Formatting and grabbing the females, males do some nuptial dance over a characteristic up-and-down pattern of movement. Afterward, the couples were released to the vegetation for mating. The mayfly optimization algorithm uses this conception for optimization [16]. This algorithm uses a hybrid conception of the particle swarm optimization (PSO) algorithm, the genetic algorithm (GA), and the firefly algorithm (FA). Based on the MFO algorithm, the initial population is divided into two classes of male and female mayflies that are generated randomly. The initial population (candidates) is considered a *d*-dimensional vector *X* = [*x*_1_, *x*_2_, ⋯,*x*_*d*_]^*T*^ which has been randomly positioned in the solution space. The mayflies have a velocity equal to *V* = [*v*_1_, *v*_2_, ⋯,*v*_*d*_]^*T*^, and their direction relates to both social and individual flying experiences. Then, the candidates tune their position close to their best position (*p*_best_), as well as the best position of the other candidates (*g*_best_).

By considering *x*_*i*_ as the present position of the candidate with a step equal *t*, the updated position is obtained by the following equation [16]:

(3) $$\begin{array}{c} x_{i}\left(t+1\right)=x_{i}\left(t\right)+v_{i}\left(t+1\right), \end{array}$$

where *x*_*i*_(0) is limited between *x*_min_ and *x*_max_.

The movement of mayflies on the top of the water to dance is mathematically modeled as follows:

(4) $$\begin{array}{c} v_{ij}\left(t+1\right)=v_{ij}\left(t\right)+a_{1}\times \exp\left(-\beta r_{p}^{2}\right)\times \left(p\text{bes}{\text{t}}_{ij}-x_{ij}^{t}\right)+a_{2}\times \exp\left(-\beta r_{g}^{2}\right)\times \left({g\text{best}}_{j}-x_{ij}^{t}\right),j=1,2,\cdots ,n \end{array}$$

where  *β* describes the visibility coefficient utilized for restraining mayfly visibility to others; *p*best_*i*_ defines the *i*^th^ best position candidate had ever visited; *r*_*p*_ and *r*_*g*_ describe the Cartesian distance between *x*_*i*_ and *p*best_*i*_ and *x*_*i*_ and *g*best; *x*_*ij*_^*t*^ and *v*_*ij*_(*t*) represent the position and the velocity of the *i*^th^ candidate in dimension *j*, respectively; and *a*_1_ and *a*_2_ signify the constants for positive attraction scaling the involvement of the social and cognitive component, respectively.

The best position for personally succeeding in the time step *t* + 1 is as follows:

(5) $$\begin{array}{c} p\text{bes}{\text{t}}_{i}=\left\{\begin{array}{c} x_{i}\left(t+1\right),\text{if }f\left(x_{i}\left(t+1\right)\right)<f\left(\;p\text{bes}{\text{t}}_{i}\right)\\ \text{is kept the same},O.W., \end{array}\right. \end{array}$$

where *f*(.) describes the objective function to define the quality of the solution. Then, the global best position (*g*best_*j*_) is achieved as follows:

(6) $$\begin{array}{c} g\text{best}=\min\left\{f\left(p\text{bes}{\text{t}}_{1}\right),f\left(p\text{bes}{\text{t}}_{2}\right),\cdots ,f\left(p\text{bes}{\text{t}}_{N}\right)\right\}, \end{array}$$

where *N* describes the total number of male candidates in the swarm. The Norm 2 equation has been utilized to determine the *r*_*p*_ and *r*_*g*_ as follows:

(7) $$\begin{array}{c} r_{p}=\sqrt{\overset{n}{\underset{j=1}{\sum }}\left(x_{ij}-p\text{bes}{\text{t}}_{i}\right),}\\ r_{g}=\sqrt{\overset{n}{\underset{j=1}{\sum }}\left(x_{ij}-g\text{best}\right),} \end{array}$$

where *x*_*ij*_ describes the *j*^th^ component of the candidate *i*.

For retaining the algorithm with the best candidates, the best mayflies keep to dance and update their velocities by the following equation:

(8) $$\begin{array}{c} v_{ij}\left(t+1\right)=v_{ij}\left(t\right)+n_{d}\times \delta , \end{array}$$

where *n*_*d*_ signifies the nuptial dance coefficient and *δ* describes a random value in the range [−1, 1].

However, each male mayfly belongs to a special swarm; the females do not belong to groups. They fly around the males for breeding. With assuming *y*_*i*_(*t*) as the *i*^th^ female candidate path, in the solution space, the position has been updated by the following equation:

(9) $$\begin{array}{c} y_{i}\left(t+1\right)=y_{i}\left(t\right)+v_{i}\left(t+1\right). \end{array}$$

The best male breeds with the best female, the second-best male with the second-best female, etc. Therefore, the velocity has been considered as follows:

(10) $$\begin{array}{c} v_{ij}\left(t+1\right)=\left\{\begin{array}{c} v_{ij}\left(t\right)+a_{2}\times \exp\left(-\beta r_{mf}^{2}\right)\times \left(x_{ij}^{t}-y_{ij}^{t}\right),\text{if }f\left(y_{i}\right)>f\left(x_{i}\right),\\ v_{ij}^{t}\left(t\right)+r_{w}\times r,f\left(y_{i}\right)\leq f\left(x_{i}\right), \end{array}\right. \end{array}$$

where *β* represents a fixed visibility coefficient, *a*_2_ describes a positive attraction constant, *r*_*mf*_ describes the Cartesian distance between male and female candidates, *y*_*ij*_^*t*^ and *v*_*ij*_^*t*^(*t*) signify the *i*^th^ female candidate position and velocity in dimension *j* at time step *t*, and *r*_*w*_ defines a random walk coefficient, and *r* is a random value in the range [−1, 1].

The MFO algorithm uses crossover as the mating process between the male and female candidates such that two candidates are first selected as male and female. The way of selecting the parent is similar to the method of female attraction by males. The new generation of the crossover process has been achieved as follows:

(11) $$\begin{array}{c} \text{offsprin}{\text{g}}_{1}=\zeta \times \text{male}+\left(1-\gamma \right)\times \text{female},\\ \text{offsprin}{\text{g}}_{2}=\zeta \times \text{female}+\left(1-\gamma \right)\times \text{male}, \end{array}$$

where *ζ* describes a random value and male and female describe the parents. And the early velocity of the offspring is set at zero.

The main reason for using the mayfly optimization algorithm is that it combines the major advantages of swarm intelligence and evolutionary algorithms, which makes it stronger in providing a good balance between exploration and exploitation [17].

### 3.1. Data Segmentation

The next step after preprocessing of the input images is to segment the pattern data. The present research uses mayfly optimization (MFO) algorithm to develop a new version of the fuzzy *C*-means technique for optimal clustering (MFO-FCM). The MFO-FCM is also a good tool for noise reduction in this application. By assuming partitioning of a set of data based on this algorithm including *N* points into *C* clusters, the best results of the fuzzy *C*-means are achieved by minimizing the following equation [18]:

(12) $$\begin{array}{c} \begin{array}{c} \min\left\{F=\overset{c}{\underset{j=1}{\sum }}\overset{N}{\underset{k=1}{\sum }}\left[a{\left(u_{jk}\right)}^{m}+b\times \kappa_{jk}{\left(t_{jk}\right)}^{\eta }\right]L\left(x_{k},v_{j}\right)+\overset{c}{\underset{j=1}{\sum }}\delta_{i}\overset{N}{\underset{k=1}{\sum }}{\left(1-t_{jk}\right)}^{\eta }\right\}\\ j=1,2,\cdots ,c;k=1,2,\cdots ,N;u_{jk}\in \left[0,1\right];\overset{c}{\underset{i}{\sum }}u_{jk}=1. \end{array}. \end{array}$$

Subject to

(13) $$\begin{array}{c} \overset{c}{\underset{j=1}{\sum }}u_{jk}=1,0\leq u_{jk};t_{jk}\leq 1;a,b>0;m,\eta >1, \end{array}$$

where *κ*_*jk*_ signifies the feature for the *j*^th^ cluster and the *k*^th^ the point, *m* signifies the weight that is set here 2, and *a* and *b* describe the effect of the variable on the status of membership and feature, respectively. In the event that *a* > *b*, membership provides more effect on the data; otherwise, the data reduces the noise effect and *L*(*x*_*k*_, *v*_*j*_) describes a value in the range *x*_*k*_ to the center of the cluster *v*_*j*_ and has been obtained by the following equation:

(14) $$\begin{array}{c} L\left(x_{k},v_{j}\right)=\sqrt{\overset{p}{\underset{l=1}{\sum }}{\left(x_{il}-v_{jl}\right)}^{2}}. \end{array}$$

As mentioned in Equation (12), the fuzzy *C*-means minimization is usually performed by the Lagrange multiplier theorem that is performed based on the following equation:

(15) $$\begin{array}{c} u_{jk}=\overset{c}{\underset{j=1}{\sum }}{\left(\frac{d\left(x_{k},v_{j}\right)}{d\left(x_{k},v_{j}\right)}\right)}^{-1/\left(m-1\right)},\\ t_{jk}=\frac{1}{1+\left[\left(b\times \beta_{jk}/\delta_{j}\right)D\left(x_{k},v_{j}\right)\right]},\\ \begin{array}{c} v_{jl}^{\left[r\right]}=\frac{\left[\sum_{k=1}^{N}\left[a{\left(u_{jk}\right)}^{m}+b\times \beta_{jk}\times {\left(t_{jk}\right)}^{\eta }\right]h_{jkl}^{\left[r\right]}x_{kl}\right]}{\left[\sum_{k=1}^{N}\left[a{\left(u_{jk}\right)}^{m}+b\times \beta_{jk}\times {\left(t_{jk}\right)}^{\eta }\right]h_{jkl}^{\left[r\right]}\right]},\\ k\in \left[1,N\right];l\in \left[1,p\right];j\in \left[1,c\right]. \end{array} \end{array}$$

In this study, the mayfly optimization (MFO) algorithm is used to generate the memberships and possibilities along with using typicality to tune the impact of the outlier. Here, the parameters *T*, *V*, and *U* are updated to minimize the considered function during the iterations. This will be terminated if ‖*V*^*r*^ − *V*^*r*−1^‖ ≤ *ε*.

The present study uses within-cluster sum of squares errors (WCSS) as the objective function for the optimization. By considering *S* = [*s*_1_, *s*_2_, ⋯, *s*_*N*_] as clusters and *X* = [*x*_1_, *x*_2_, ⋯, *x*_*N*_] as data points, the WCSS function is formulated by the following equation:

(16) $$\begin{array}{c} \text{WCSS}=\overset{k}{\underset{j=1}{\sum }}\underset{x_{j}\in s_{i}}{\sum }L\left(x_{j},v_{i}\right), \end{array}$$

where *L*(*x*_*j*_, *v*_*i*_) determines the distance from *v*_*i*_ and *x*_*j*_, and *v*_*i*_ represents the center of the cluster *s*_*i*_.

This method contains two parts: the first part is to identify the model parameters and the second part is to centroid clustering. Six variables are considered in the MFO-FCM model. By assuming cluster centroids including *V* = [*v*_1_, *v*_2_, ⋯, *v*_*c*_], the centers of the clusters are defined. The pseudocode of the MFO-FCM model is explicated in the following:

### 3.2. Enhanced Capsule Networks (ECN)

The present study uses enhanced capsule networks (ECN) to provide a suitable diagnosis system. In ECN, the fragmented pixel set of the X-ray image is labeled as a set of nerve cells regarding the capsule. The system used a pixel vector as an actuation vector encompassed by an active capsule such that it may be a particular category like healthy or COVID-19 for the X-ray image.

Here, the capsule output and the coupling coefficient have been multiplied by the capsule routing in a layer. The parent capsule resistance for routing defines the value of the coupling coefficient. Based on the *routing-by-agreement* mechanism, the low-level COVID-19 diagnosis has been determined by the high-level capsule activation [19].

With assuming *y*_*i*_ ∈ [healthy, COVID‐19] as the *i*^th^ output capsule, and *we*_*ij*_ as the weight matrix, we have

(17) $$\begin{array}{c} {\hat{y}}_{\left(j\;|\;i\right)}=we_{ij}y_{i}, \end{array}$$

where ${\hat{y}}_{\left(j|i\right)}$ signifies the detection vector which identifies the output parent capsule *j* using capsule *i*.

The value of the weight will be amended if the values will be reduced or the pixels contain probably to the positive class. For going before layer capsules, the softmax function has been utilized and the potential parent capsule as the coefficient is encrypted *c*_*ij*_ such that essential logits *b*_*ij*_ appears the log past conceivable outcomes of the *i*^th^ routing capsule within the last layer to the *j*^th^ capsule within the succeeding layer.

The routing-by-agreement mechanism is mathematically performed by the following equation:

(18) $$\begin{array}{c} c_{ij}=\frac{e^{b_{ij}}}{\sum e^{b_{ij}}}. \end{array}$$

And the proceeding layer indicates a critical element in the evaluation of input of the parent capsule *j* as follows:

(19) $$\begin{array}{c} s_{j}=\underset{i}{\sum }c_{ij}{\hat{y}}_{\left(j\;|\;i\right)}. \end{array}$$

The squashing function has been utilized to accomplish the pixel vector compression in the range [0, 1) as follows:

(20) $$\begin{array}{c} va_{j}=\frac{{\left\|s_{j}\right\|}^{2}}{1+{\left\|s_{j}\right\|}^{2}}\times \frac{s_{j}}{\varepsilon +{\left\|s_{j}\right\|}^{2}}, \end{array}$$

where *ε* = 10^−7^.

And the capsule in the next layer is formulated by the following equation:

(21) $$\begin{array}{c} a_{ij}=va_{j}\times {\hat{y}}_{\left(j\;|\;i\right)}. \end{array}$$

The overall classification of the capsules which are considered as individual margin loss (Loss_*k*_) in the categories capsule *k* for the capsule networks based on the loss is

(22) $$\begin{array}{c} \text{Los}{\text{s}}_{k}=T_{k}\max{\left(0,m^{+}-\left\|{va}_{k}\right\|\right)}^{2}+\lambda \left(1-T_{k}\right)\max{\left(0,\left\|{va}_{k}\right\|-m^{-}\right)}^{2}, \end{array}$$

where *T*_*k*_ describes the instant attendance in category capsule *k* and *m*^−^, *m*^+^, and *λ* represent hyperparameter assistances. Finally, the ECN has been trained by 700 iterations using Adam optimizer to get the optimal results of hyperparameters. The learning rate is considered an amount of 1*e* − 6 [20].

## 4. Results and Discussions

The proposed model is performed on three datasets including a popular resource collected by the Renmin Hospital of Wuhan University and two affiliated hospitals, Sun Yat-sen Memorial Hospital and the Third Affiliated Hospital of the Sun Yat-sen University in Guangzhou with 12 and 76 patients [10]. The method is programmed in MATLAB 2016b 64-bit version and executed on the computation environment of Intel Core i7 CPU 2.00 GHz, 2.5 GHz, 8 GB RAM, and 64-bit operating system. As before mentioned, the key purpose of this study is to design a new CAD-based system for the diagnosis of COVID-19. The model has been analyzed based on four measurement indicators including accuracy, precision, *F*1-score, and sensitivity.

### 4.1. Accuracy

The precision determines how nearly the measured esteem is to the genuine (real) value. This indicator is accomplished by the proportion of correct identification values to the whole number of identifications. This can be mathematically described as follows:

(23) $$\begin{array}{c} \text{Accuracy}=\frac{\sum_{i=1}^{l}\left(\text{T}{\text{P}}_{i}+\text{T}{\text{N}}_{i}\right)}{\sum_{i=1}^{\text{l}}\left(\text{T}{\text{P}}_{i}+\text{T}{\text{N}}_{i}+\text{F}{\text{P}}_{i}+\text{F}{\text{N}}_{i}\right)}, \end{array}$$

where TN and FN describe the true negative and false negative, respectively, and TP and FP represent the true positive and false positive, respectively.

### 4.2. Precision

Precision defines how near the measured values are to each other. This indicator is accomplished by the proportion of positive identification values to the whole number of identifications. This can be mathematically described as follows:

(24) $$\begin{array}{c} \text{Precision}=\frac{\sum_{i=1}^{l}\left(\text{T}{\text{P}}_{i}+{\text{FP}}_{i}\right)}{\sum_{i=1}^{l}\left(\text{T}{\text{P}}_{i}+\text{T}{\text{N}}_{i}+\text{F}{\text{P}}_{i}+\text{F}{\text{N}}_{i}\right)}. \end{array}$$

### 4.3. Sensitivity

Sensitivity is the extent of positives that are accurately recognized (i.e., the extent of those who have a few conditions (influenced) who are accurately recognized as having the condition). This indicator is accomplished by the proportion of true identification values to the true positive and false negative number of identifications. This can be mathematically described as follows:

(25) $$\begin{array}{c} \text{Recall}=\frac{\sum_{i=1}^{l}\text{T}{\text{P}}_{i}}{\sum_{i=1}^{l}\left(\text{T}{\text{P}}_{i}+\text{F}{\text{N}}_{i}\right)}. \end{array}$$

### 4.4. *F*1-Score

*F*-score or *F*-measure could be the degree of a test's exactness. It is achieved based on the precision and sensitivity of the test. The most noteworthy conceivable value of an *F*-score is 1.0, showing idealized exactness and review, and the least conceivable value is 0, with the chance that either the precision or the sensitivity is zero. The *F*1-score is additionally known as the Dice similarity coefficient (DSC). This measure is mathematically defined as follows:

(26) $$\begin{array}{c} F1_{\text{score}}=\frac{2\times \text{Precision}\times \text{Recall}}{\text{Precision}+\text{Recall}}. \end{array}$$

The method analysis of the classification based on the offered enhanced capsule networks (ECN) is reported in Tables 1–4. The method has been compared with three other state-of-the-art methods including FOMPA [21], MID [22], and 4S-DT [23] for better clarification.

**Figure 4:**
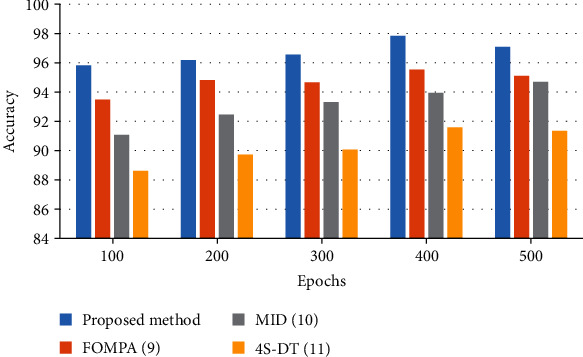
The bar plot of the accuracy results for the studied algorithms.

**Figure 5:**
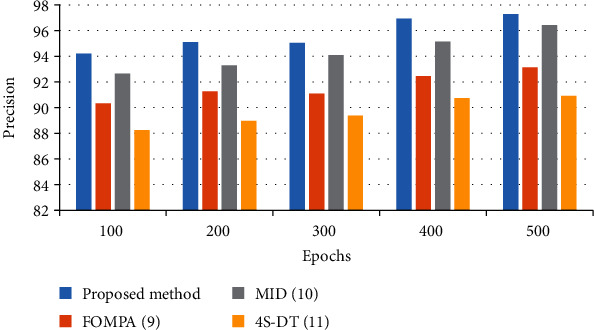
The bar plot of the precision results for the studied algorithms.

**Figure 6:**
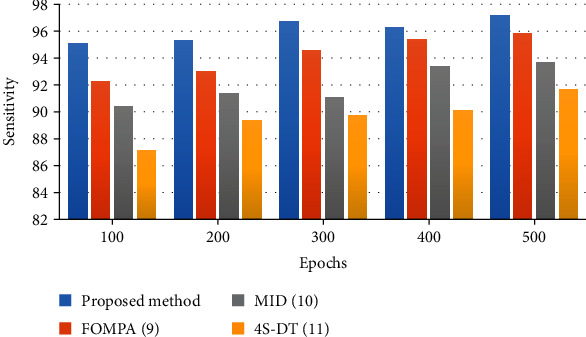
The bar plot of the sensitivity results for the studied algorithms.

**Figure 7:**
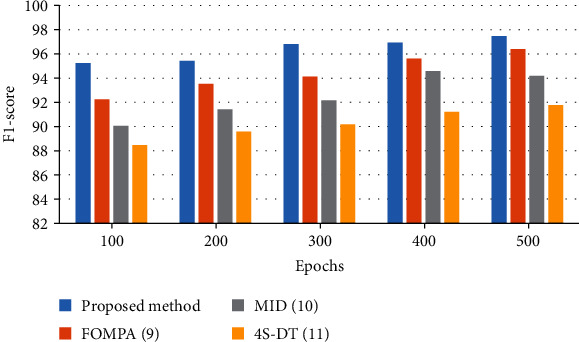
The bar plot of the *F*1-score results for the studied algorithms.

To offer a better clarification of the efficiency of the proposed method, a bar plot of the results is shown in Figures 4–7. As can be observed from Figure 4, the simulation shows a 97.08% accuracy with 97.29% precision for the suggested methodology compared to the other studied methods. However, FOMPA, MID, and 4S-DT are in the next ranks.

Figure 5 shows the precision results for the studied algorithms.

Figure 6 shows the bar plot of the sensitivity results for the studied algorithms.

The results show that 500 epochs for all algorithms have been utilized. As can be observed from Figure 6, the proposed classifier offers a higher sensitivity rate to the other state-of-the-art. The designed classifier of the proposed method offers a 97.1% sensitivity rate, whereas FOMPA, MID, and 4S-DT have 95.85%, 93.66%, and 91.68%, respectively, for 500 epochs.  Figure 7 illustrates the bar plot of the *F*1-score results for the studied algorithms.

Based on Figure 7, after 500 epochs for all algorithms, the proposed method presents the best better *F*1-score rate to the other methods. As can be observed, the suggested method with a 97.47% *F*1-score rate has the highest value, and the FOMPA, MID, and 4S-DT with 96.39%, 94.18%, and 91.76%, respectively, are in the next ranks.

## 5. Conclusions

COVID-19 was formed in late 2019 and is spreading rapidly across the world. Early diagnosis of COVID-19 can be so beneficial to the treatment of the disease and to prevent its outbreak. Due to the probability of human error among the experts in finding COVID-19, the application of machine learning has been recently increased as an auxiliary tool. The present study proposed a method based on image processing for the diagnosis of COVID-19. This study presented an optimal configuration for proper diagnosis of COVID-19 based on an optimized fuzzy *C*-ordered means (FCOM) and an improved version of the enhanced capsule network (ECN). The ECN was improved based on the mayfly optimization (MFO) algorithm. The proposed method was then performed on the chest X-ray COVID-19 images from publicly available datasets. The results were analyzed by comparing some other methods, including FOMPA, MID, and 4S-DT, and the results showed the higher effectiveness of the proposed method. As mentioned, the proposed method has good accuracy in terms of theory. However, due to using complicated methods, using it in real-time applications is not feasible. Therefore, in future work, we will work on using a simplified technique of the proposed method to perform on a microprocessor for real-time applications.

## Figures and Tables

**Pseudocode 1:**
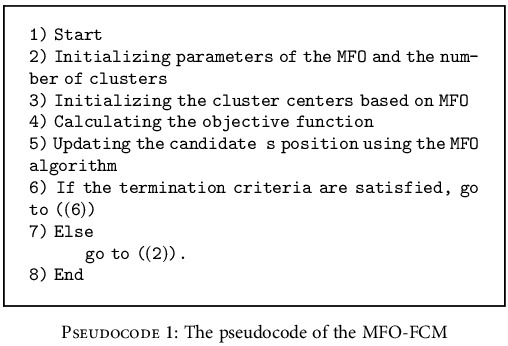
The pseudocode of the MFO-FCM

**Table 1:** Accuracy results based on different methods.

Epochs	Proposed method	FOMPA [21]	MID [22]	4S-DT [23]
100	95.82	93.49	91.08	88.61
200	96.19	94.82	92.46	89.73
300	96.55	94.67	93.32	90.07
400	97.83	95.53	93.95	91.59
500	97.08	95.11	94.70	91.34

**Table 2:** The precision results based on different methods.

Epochs	Proposed method	FOMPA [21]	MID [22]	4S-DT [23]
100	94.20	90.33	92.64	88.24
200	95.11	91.27	93.29	88.96
300	95.05	91.08	94.08	89.38
400	96.93	92.46	95.13	90.73
500	97.29	93.14	96.42	90.91

**Table 3:** The sensitivity results based on different methods.

Epochs	Proposed method	FOMPA [21]	MID [22]	4S-DT [23]
100	95.05	92.26	90.43	87.15
200	95.31	93.04	91.38	89.36
300	96.73	94.53	91.07	89.75
400	96.29	95.38	93.40	90.07
500	97.18	95.85	93.66	91.68

**Table 4:** *F*1-score results based on different methods.

Epochs	Proposed method	FOMPA [21]	MID [22]	4S-DT [23]
100	95.23	92.25	90.05	88.46
200	95.42	93.53	91.42	89.57
300	96.81	94.12	92.16	90.18
400	96.93	95.60	94.57	91.22
500	97.47	96.39	94.18	91.76

## Data Availability

The presented study uses three datasets including a popular resource collected by the Renmin Hospital of Wuhan University and two affiliated hospitals, Sun Yat-sen Memorial Hospital and the Third Affiliated Hospital of the Sun Yat-sen University in Guangzhou, that can be achieved by email to the sources.
